# Pattern evolution of antidepressants and benzodiazepines use in a cohort

**DOI:** 10.11606/s1518-8787.2020054001887

**Published:** 2020-03-31

**Authors:** Geisy de Carvalho Alcantara, Evandro Silva Freire Coutinho, Eduardo Faerstein

**Affiliations:** I Universidade do Estado do Rio de Janeiro Instituto de Medicina Social Programa de Pós-Graduação em Saúde Coletiva Rio de JaneiroRJ Brasil Universidade do Estado do Rio de Janeiro. Instituto de Medicina Social. Programa de Pós-Graduação em Saúde Coletiva. Rio de Janeiro, RJ, Brasil; II Universidade do Estado do Rio de Janeiro Instituto de Medicina Social Departamento de Epidemiologia Rio de JaneiroRJ Brasil Universidade do Estado do Rio de Janeiro. Instituto de Medicina Social. Departamento de Epidemiologia. Rio de Janeiro, RJ, Brasil

**Keywords:** Benzodiazepines, administration & dosage, Antidepressive Agents, administration & dosage, Drug Utilization, trends, Cohort Studies

## Abstract

**OBJECTIVE:**

In recent decades there has been an increase in the use of antidepressants (AD) and a decrease in the use of benzodiazepines (BDZ). Prevalence, cumulative incidence, and factors associated with the incidence of AD and BDZ use in a Brazilian population were estimated in this article.

**METHODS:**

Data were collected with a self-administered questionnaire in a cohort of employees from a university in Rio de Janeiro. The prevalence of the use of AD and BDZ was calculated for 1999 (4,030), 2001 (3,574), 2006-07 (3,058), and 2012 (2,933). The cumulative incidences of the use of AD and BDZ between 1999 and 2007 were estimated by the Poisson models with robust variance estimates.

**RESULTS:**

In 1999, the prevalence of the use of AD and BDZ were 1.4% (95%CI: 1.1–1.8) and 4.7% (95%CI: 4.1–5.4), respectively; in 2012, they were 5.4% (95%CI: 5.5–6.2) and 6.8% (95%CI: 6.0–7.8). The incidence of use, between 1999 and 2007, was 4.9% (95%CI: 4.2–5.7) for AD and 8.3% (95%CI: 7.3–9.3) for BDZ. The incidences of AD and BDZ use were higher among women and participants with a positive General Health Questionnaire.

**CONCLUSION:**

In this population, the increase in the use of AD was not accompanied by a decrease in the use of BDZ, showing the prescriptions for psychotropic medication do not follow the currently recommended guidelines for treatment of common mental health disorders.

## INTRODUCTION

Antidepressants (AD) and benzodiazepines (BDZ) are the most used psychotropic medications. The first AD was introduced in the 1950s and presented important side effects and contraindications, while BDZ showed fewer side effects and were considered to be safer, resulting in their more frequent prescription by psychiatrists and non-psychiatrists alike. With the introduction of fluoxetine, a selective serotonin reuptake inhibitors (SSRI) in the 1980s with fewer side effects, the AD use increased more than the observed for BDZ^1 , 2^ . International guidelines recommend the use of BDZ should be restricted to patients with major depression disorders associated with anxiety and/or insomnia, and only if the AD did not provide an adequate treatment^2 , 3^ .

In 1988, in the United States (US), 9.7% of patients with a depression diagnosis received a prescription for the SSRI fluoxetine. This proportion gradually rose to 21% (1989), 46% (1993), and 69% (2001). In parallel, there was a decrease in the use of BDZ for depression treatment: in 1987, 21% of individuals used BDZ for this purpose, but in 2001 the proportion was only 7.5%^1^ .

There is significant variation in the prevalence of use of AD and BDZ in the general population among countries, for example 2.9% (AD) and 3.8% (BDZ) in Chile in 1996–98^4^ ; 4.7% (AD) and 11.4% (BDZ) in Spain in 2001–02^5^ ; and 7.2% (AD) and 12.3% (BDZ) in Belgium in 2001–02^6^ . A study conducted in 2000 in six European countries (Belgium, France, Germany, Italy, Netherlands and Spain), estimated 3.7% annual prevalence for AD and 9.8% for BDZ^7^ .

In Brazil, a study in 1988 among 1,459 residents in Rio de Janeiro found a 5.2% prevalence for the use of psychotropic medicine in the previous month, 85% being BDZ^8^ . Around 20 years later, in another study in Rio^9^ , the most used type of psychotropic medicines in the previous month was the AD, with 2.8%, and the prevalence of BDZ use was 1.6%. Several studies have been conducted in São Paulo. In 1990, among 1,742 individuals, only five had used AD in the previous 12 months, compared with the 140 that had used BDZ, a prevalence of 8%^10^ . Later (2007), one study estimated similar prevalence of BDZ (3.7%) and AD (3.5%) use in the previous 12 months^11^ , and in another one the prevalence of AD use in the previous 30 days was 3.1%, and BDZ use was 2.7%^12^ . Studies point to greater use of psychotropic medicine by women, older adults, those with a higher income and education (especially in the AD case), the unemployed, and people who are separated or divorced^2 , 9 , 11^ . Some findings are inconsistent as some studies report, for example, a higher prevalence among less educated individuals^2 , 8^ .

The objective of this study is to investigate the prevalence and cumulative incidence of AD and BDZ among Brazilian adults and factors associated with the use of these medicines. This study hypothesizes that the use of AD has been increasing since the year 2000 while the use of BDZ has been decreasing.

## METHODS

### Study design

A concurrent cohort study with data from the Pró-Saúde Study, a longitudinal study of university civil servants in Rio de Janeiro^15^ .

The wave 1 of the study (baseline) occurred in 1999, with 4,030 participants (91% of those eligible); wave 2 occurred in 2001–2 (3,574 individuals, 83% of those eligible); wave 3 occurred in 2006-7, with 3,058 individuals (76% of those eligible); wave 4 occurred in 2012, with 2,933 individuals (58% of those eligible)^15^ .

The ethics committee of the Universidade Estadual do Rio de Janeiro approved the research protocol; all subjects signed an informed consent form.

### Variables

The variables used in the study were obtained with a self-administered questionnaire applied during wave 1 (1999):

- Sociodemographic (sex, age, marital state, self-reported race/skin color, education level, and equivalent income compared with minimum wage at the time).
- Use of alcohol. This variable was assessed by three questions: i. “ *Did you consume any type of alcoholic drink in the last two weeks?* ” ii. “ *How many days did you consume any type of alcoholic drink in the last two weeks?* ” iii. “ *How many doses did you consume in the days you drank in the last two weeks?* ”
- Self-reported health assessment (general health condition and current health condition).
- Sleep problems (difficulty in initiating sleep and waking up frequently at night).
- Tiredness for no apparent reason.
- Mental health was assessed with the Brazilian version of the General Health Questionnaire – GHQ-12 (validated by a structured psychiatric interview)^16^ during waves 1, 2, and 3 of the study. The GHQ-12 scores were dichotomized, using scores from 3 or more as indicative of common mental disorder (CMD).
- The use of psychotropic drugs was assessed with the construction of a dichotomous variable (no/yes) based on a question referring to medicine use: *“During the past two weeks, have you used any medicine? If the answer is yes, which medicine have you used in the past two weeks?”* . During waves 3 and 4, the information about medicine use regarded the previous seven days.

The participants were classified as AD or BDZ users if reported (i) the name of the substance or (ii) the pharmacological class. If the name of the medicine was not reported, the Anatomical Therapeutic Chemical (ATC) classification was used^17^ .

### Analysis

A descriptive analysis of the study population was performed with measures estimations of central tendency and dispersion for the continuous variables and frequency distribution for the categorical variables. Prevalence and 95% confidence interval (CI) were estimated for the use of AD and BDZ.

Then a fixed cohort with individuals who did not use AD or BDZ during wave 1 was assembled and observed until wave 3, thus providing eight years of follow-up. Cumulative incidences of AD and BDZ were estimated in both phases following wave 1 of the study. Wave 4 was excluded from the follow-up analysis due to many study participants who quit the follow-up. In an exploratory data analysis, incidences were estimated and stratified by sociodemographic, general, and mental health variables. The initial choice of variables among the ones collected in the original study considered the relationship of the variables with the use of AD and BDZ present in the literature. The associations were considered statistically significant if p-value ≤ 0.05, and of borderline statistical significance if between 0.06 and 0.10.

To estimate the cumulative incidence ratio (relative risk) the Poisson regression models with robust variance estimates were adjusted, having as a dependent variable the incidence of AD and BDZ use. Initially, the models were adjusted with only one independent variable at a time. Variables associated with p-values ≤ 0.20 were selected for subsequent assessment in the multivariate models, with their inclusion one by one; those associated with p *-* values > 0.10 were kept in the model.

The analysis was performed using the statistical software *Stata* version 12.

## RESULTS

### Population characteristics during wave 1 (baseline)

The overall sample in wave 1 contained 4,030 individuals. Most of them were female (55%), ranging from 35 to 44 years of age (43%). Most participants were married/cohabiting (60%), self-reported as being white (52%), who completed higher education (27%), and worked at the university hospital (49%). Two-thirds of the individuals had an income greater than six times the minimum wage. More than half the individuals (56%) reported having private health insurance ( Table 1 ).

**Table 1:** Sociodemographic characteristics, self-reported health conditions, and prevalence of antidepressants and benzodiazepines use at the baseline (1999). Pró-Saúde Study, Brazil.

	Baseline (4,030) N (%)	Prevalence of AD % (95%CI)	p-value	Prevalence of BDZ % (95%CI)	p-value
Total		1.4 (1.1–1.8)		4.7 (4.0–5.4)	

Sex					
Men	1,792 (44.5)	0.5 (0.2–0.9)	< 0.001	2.8 (2.1–3.6)	< 0.001
Women	2,238 (55.5)	2.1 (1.5–2.7)		6.2 (5.2–7.2)	
Age					
22–34	1,124 (27.9)	1.3 (0.7–2.0)	0.922	2.2 (1.4–3.1)	< 0.001
35–44	1,740 (43.1)	1.4 (0.8–1.9)		5.0 (4.0–6.1)	
45–54	885 (22.0)	1.6 (0.7–2.4)		7.0 (5.3–8.7)	
55 or more	281 (7.0)	1.8 (0.2–3.3)		5.3 (2.7–8.0)	
Marital status					
Married or living together	2,397 (59.5)	1.0 (0.6–1.4)	0.06	4.1 (3.3–4.9)	< 0.001
Separated or divorced	611 (15.0)	2.1 (1.0–3.3)		8.3 (6.1–10.5)	
Widower	116 (3.0)	2.6 (0.0–5.5)		6.9 (2.3–11.5)	
Single	805 (20.0)	2.0 (1.0–2.9)		3.5 (2.2–4.7)	
No information	101 (2.5)	–		–	
Race					
Black	626 (15.5)	1.4 (0.5–2.4)	0.811	4.9 (3.2–6.6)	0.576
Brown	1,171 (29.1)	1.4 (0.8–2.1)		4.4 (3.2–5.6)	
White	2,082 (51.7)	1.5 (1.0–2.1)		5.0 (4.0–5.9)	
Asian	60 (1.5)	–		3.3 (–1.2–7.9)	
Indigenous	41 (1.0)	–		–	
No information	50 (1.2)	–		–	
Education level					
Some elementary school	276 (6.8)	1.1 (–0.1–2.3)	0.951	5.1 (2.5–7.7)	0.609
Elementary school	305 (7.6)	1.6 (0.2–3.1)		3.9 (1.7–6.1)	
Some high school	365 (9.0)	1.4 (0.2–2.6)		6.6 (4.0–9.1)	
High school	870 (21.6)	1.6 (0.8–2.4)		4.8 (3.4–6.2)	
Some college	560 (14.0)	1.2 (0.3–2.2)		3.7 (2.2–5.3)	
College	1,084 (27.0)	1.3 (0.6–2.0)		4.7 (3.4–6.0)	
Graduate	523 (13.0)	1.9 (0.7–3.1)		4.6 (2.8–6.4)	
No information	47 (1.0)	–		–	
Workplace					
UERJ^1^ Campus	2,053 (51.0)	1.2 (0.7–1.7)	0.229	4.0 (3.2–4.9)	0.04
HUPE^2^ Hospital	1,977 (49.0)	1.7 (1.1–2.2)		5.4 (4.4–6.4)	
Income (equal to number of minimum wages)					
Up to 3	329 (8.7)	1.2 (0.02–2.4)	0.6	5.7 (3.2–8.3)	0.619
3 to 6	967 (25.5)	1.1 (0.5–1.8)		4.4 (3.1–5.7)	
> 6	2,490 (65.8)	1.6 (1.1–2.0)		4.7 (3.9–5.6)	
Health insurance users					
Yes	2,266 (56.2)	1.5 (0.9–2.0)	0.638	4.9 (4.0–5.9)	0.446
No	1,739 (43.1)	1.3 (0.8–1.8)		4.4 (3.4–5.4)	
No information	25 (0.6)	–		–	
Alcohol consumption					
Yes	2,148 (53.3)	1.1 (0.6–1.5)	0.03	3.9 (3.1–4.8)	0.01
No	1,751 (43.5)	1.9 (1.2–2.5)		5.7 (4.6–6.8)	
No information	131 (3.2)	–		–	
Frequency of alcohol consumption (2 weeks)					
Every day	84 (2.1)	1.2 (0.0–3.5)	0.73	5.9 (0.8–11.0)	0.76
10 to 13 days	77 (1.9)	–		2.6 (0.0–6.2)	
6 to 9 days	172 (4.3)	0.6 (0.0–1.7)		3.5 (0.7–6.2)	
2 to 5 days	1.098 (27.2)	1.0 (0.4–1.6)		4.2 (3.0–5.4)	
1 single day	708 (17.6)	1.4 (0.5–2.3)		3.5 (2.2–4.9)	
Amount of alcohol consumed per day (2 weeks)					
1 standard unit	631 (15.7)	1.1 (0.3–1.9)	0.98	3.2 (1.8–4.5)	0.3
2 to 4 standard unit	902 (22.4)	1.1 (0.4–1.8)		4.1 (2.8–5.4)	
5 to 7 standard unit	311 (7.7)	1.0 (0.0–2.0)		3.2 (1.2–5.2)	
8 a 10 standard unit	141 (3.5)	1.4 (0.0–3.4)		5.0 (1.4–8.6)	
More than 10 standard unit	131 (3.2)	0.8 (0.0–2.3)		6.9 (2.5–11.2)	
Health condition					
Very good	1,132 (28.1)	0.4 (0.05–0.8)	< 0.001	1.8 (1.0–2.5)	< 0.001
Good	2,131 (52.9)	1.2 (0.7–1.6)		4.3 (3.4–5.1)	
Regular	683 (16.9)	3.2 (2.0–4.5)		9.9 (7.7–12.2)	
Bad	63 (1.6)	9.5 (2.2–16.8)		14.3 (5.6–23.0)	
No information	21 (0.5)	–		–	
Current health condition					
Better than 12 months ago	784 (19.5)	2.4 (1.3–3.5)	< 0.001	5.3 (3.8–6.9)	< 0.001
Same as 12 months ago	2,721 (67.5)	0.9 (0.5–1.2)		3.3 (2.6–4.0)	
Worse than 12 months ago	501 (12.4)	3.0 (1.5–4.5)		11.4 (8.6–14.2)	
No information	24 (0.6)	–		–	
GHQ-12 (positive ≥ 3)					
Negative	2,740 (68.0)	0.7 (0.4–1.0)	< 0.001	2.3 (1.8–2.9)	< 0.001
Positive	1,233 (30.6)	3.1 (2.1–4.0)		10.1 (8.4–11.8)	
No information	57 (1.4)	–		–	
Difficulty falling asleep					
Always	178 (4.4)	5.0 (1.8–8.3)	< 0.001	23.6 (17.3–29.8)	< 0.001
Almost Always	297 (7.4)	5.5 (2.5–7.5)		14.5 (10.5–18.5)	
Sometimes	933 (23.1)	1.7 (0.9–2.5)		5.5 (4.0–7.0)	
Rarely	887 (22.0)	0.8 (0.2–1.4)		2.7 (1.6–3.8)	
Never	1,699 (42.2)	0.6 (0.2–1.0)		1.5 (0.9–2.1)	
No information	36 (0.9)	–		–	
Wake up during sleep cycle					
Always	139 (3.5)	4.3 (0.9–7.7)	< 0.001	23.7 (16.6–30.8)	< 0.001
Almost always	320 (7.9)	3.7 (1.7–5.8)		11.2 (7.8–14.7)	
Sometimes	902 (22.4)	2.0 (1.1–2.9)		6.8 (5.1–8.4)	
Rarely	915 (22.7)	1.5 (0.7–2.3)		3.7 (2.5–4.9)	
Never	1,723 (42.7)	0.5 (0.1–0.8)		1.4 (0.8–1.9)	
No information	31 (0.8)	–		–	
Tiredness for no apparent reason					
Always	191 (4.7)	4.2 (1.3–7.0)	< 0.001	16.2 (11.0–21.5)	< 0.001
Almost always	423 (10.5)	4.5 (2.5–6.5)		10.2 (7.3–13.0)	
Sometimes	1,107 (27.5)	1.5 (0.8–2.3)		5.5 (4.2–6.8)	
Rarely	765 (19.0)	1.0 (0.3–1.8)		2.9 (1.7–4.1)	
Never	1,504 (37.3)	0.3 (0.0–0.6)		1.9 (1.2–2.6)	
No information	40 (1.0)	–		–	

^1^ Universidade Estadual do Rio de Janeiro

^2^ Hospital University Pedro Ernesto

Almost half the participants classified their health as good, and two-thirds considered their current health condition to be the same as 12 months before. Mental health assessment with the GHQ-12 showed 31% met the criteria for CMD. Two-thirds of the individuals said they never or rarely had trouble falling asleep. Also they never or rarely woke up during the sleep cycle. More than half the participants (56%) never or rarely declared tiredness for no reason ( Table 1 ).

### Prevalence of antidepressant and benzodiazepine use

The prevalence of AD and BDZ use was higher among women. The greatest difference was in AD use, with women presenting a prevalence of approximately four times higher than men ( Table 1 ).

Overall, the prevalence of AD use increased with age. As for the BDZ, there was an increase in usage by people between 45 and 54 years old. The prevalence of both AD and BDZ use was lower in married individuals, and for BDZ it was also lower in the single group ( Table 1 ).

The prevalence of AD and BDZ use lacked distinctions by race/color, education level, income, and possession of private health insurance. Regarding the workplace, the only difference in prevalence was in BDZ use, which was 35% higher among participants at the university hospital ( Table 1 ).

Increased use of AD and BDZ was noticed when the individual had a worse health perception. Among individuals with CMD, the prevalence of AD and BDZ use was close to four times higher. There was an increasing gradient of AD and BDZ use with the intensity of sleep complaints or with tiredness for no apparent reason ( Table 1 ).

**Figure 1:**
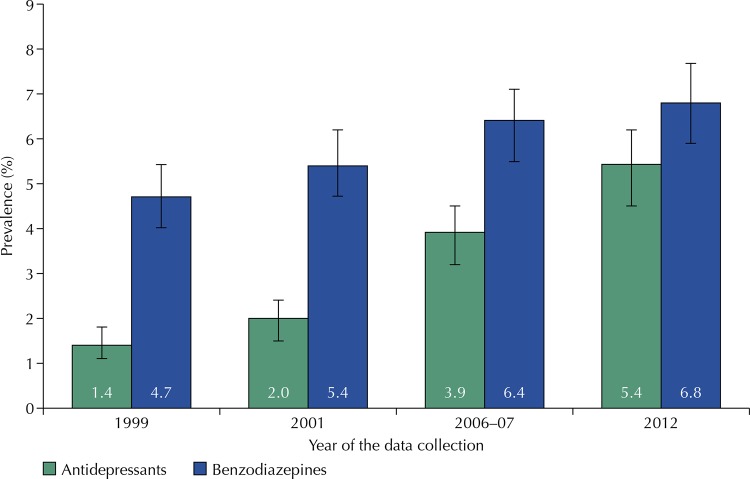
Prevalence of antidepressants and benzodiazepines in the four cohort waves (1999, 2001, 2006-07, 2012). Pró-Saúde Study, Brazil. 1999 and 2001: two-weeks prevalence; 2006-2007 and 2012: one-week prevalence

In 1999, the prevalence of AD use in the 15 previous days was reported by 1.4% of participants (95%CI: 1.1–1.8); in 2012, even over a smaller span (the previous seven days), the prevalence was 5.4% (95%CI: 4.6–6.2). As for the prevalence of BDZ, there was a progressive, although discreet, increase in use with an apparent tendency to stability: being 4.7% (95%CI: 4.1–5.4) in 1999 and 6.8% (95%CI: 6.0–7.8) in 2012 ( Figure 1 ).

In 1999, 58 individuals reported using AD in the previous two weeks. Tricyclic antidepressants (TCA), which represented in 1999 60.3% of the used AD, decreased to 15.2% in 2012. In contrast, SSRI boosted the increase in AD use, from 29.3% in 1999 to 67.6% in 2012, being primarily responsible for this increase. In parallel, there was a significant decrease in the use of bromazepam, from 43.7% in 1999 to 12.0% in 2012 ( Table 2 ).

**Table 2:** Distribution of antidepressants and benzodiazepines users according to class and medications (1999, 2001, 2006-2007, 2012). Pró-Saúde Study, Brazil.

Classes/medications	Phase 1 N (%)	Phase 2 N (%)	Phase 3 N (%)	Phase 4 N (%)
Antidepressants				

Non-selective monoamine inhibitors	35 (60.3)	26 (36.7)	31 (22.2)	24 (15.2)
Amitriptyline	17 (29.3)	12 (17.0)	14 (10.0)	12 (7.6)
Amitriptyline + chlordiazepoxide	4 (6.9)	6 (8.5)	5 (3.6)	0.0
Clomipramine	2 (3.4)	3 (4.2)	6 (4.3)	3 (1.9)
Imipramine	9 (15.5)	4 (5.6)	1 (0.7)	4 (2.5)
Nortriptyline	3 (5.2)	1 (1.4)	5 (3.6)	5 (3.2)
Selective Serotonin Reuptake Inhibitors	17 (29.31)	32 (45.1)	87 (62.7)	106 (67.6)
Citalopram	1 (1.7)	0.0	3 (2.2)	10 (6.4)
Escitalopram	0.0	0.0	4 (2.9)	12 (7.6)
Fluoxetine	10 (17.2)	17 (24.0)	51 (36.7)	47 (30.0)
Paroxetine	3 (5.2)	11 (15.5)	13 (9.4)	16 (10.2)
Sertraline	3 (5.2)	4 (5.6)	16 (11.5)	21 (13.4)
Others	3 (5.1)	3 (4.2)	16 (11.5)	22 (13.9)
Bupropion	0.0	3 (4.2)	4 (2.9)	3 (1.9)
Desvenlafaxine	0.0	0.0	0.0	1 (0.6)
Duloxetine	0.0	0.0	2 (1.4)	6 (3.8)
Mirtazapine	1 (1.7)	0.0	1 (0.7)	0.0
Trazodone	1 (1.7)	0.0	4 (2.9)	4 (2.5)
Venlafaxine	1 (1.7)	0.0	5 (3.6)	8 (5.1)
Only the class declared	4 (6.9)	10 (14.1)	7 (5.0)	6 (3.8)

Benzodiazepines				

Alprazolam	8 (4.2)	24 (12.4)	28 (12.2)	21 (10.5)
Bromazepam	83 (43.7)	66 (34.0)	43 (18.8)	24 (12.0)
Clobazam	2 (1.0)	0.0	2 (0.9)	3 (1.5)
Clonazepam	9 (4.7)	21 (10.8)	82 (36.0)	101 (50.5)
Clorazepate dipotassium	1 (0.5)	0.0	0.0	0.0
Cloxazolam	16 (8.4)	23 (11.9)	15 (6.5)	15 (7.5)
Diazepam	41(21.6)	38 (19.6)	44 (19.2)	24 (12.0)
Flunitrazepam	0.0	10 (0.5)	5 (2.2)	2 (1.0)
Flurazepam	3 (1.6)	2 (1.0)	1 (0.4)	2 (1.0)
Lorazepam	4 (2.1)	6 (3.1)	2 (0.9)	2 (1.0)
Midazolam	3 (1.6)	4 (2.1)	3 (1.3)	2 (1.0)
Nitrazepam	0.0	1 (0.5)	0.0	0.0
Only the class declared	23 (12.1)	13 (6.7)	10 (4.4)	8 (4.0)

One individual may be in more than one drug pharmacological class.

### Incidence of antidepressant and benzodiazepine use

There were increases in AD and BDZ use throughout the study period. The incidence of AD in 2001 was 1.5%, and 3.4% for BDZ. For the years of 2006-7, the cumulative incidence of eight years after wave 1 (baseline) were 3.6% for AD and 5.0% for BDZ.

The incident cases in both waves after the baseline totaled 150 individuals (4.9%) for AD and 243 (8.3%) for BDZ. The incidence among women was 3.4 times higher for AD and twice as high for BDZ than among men. Individuals aged 55 years or older used AD the least (1.7%); the opposite was found in the incidence of BDZ (13.3%). Widowers were those who most started using AD (7.4%) and BDZ (22.2%) in the follow-up period. ( Table 3 ).

**Table 3:** Cumulative incidence over 8 years (1999-2007) of antidepressant and benzodiazepine use stratified according to sociodemographic variables and self-reported health condition. Pró-Saúde Study, Brazil.

	Incidence of AD (%) (95%CI)	p-value	Incidence of BDZ (%) (95%CI)	p-value
Total	4.9 (4.2–5.7)		8.3 (7.3–9.3)	

Sex				
Men	2.1 (1.3–2.9)	< 0.001	5.3 (4.1–6.6)	< 0.001
Women	7.2 (6.0–8.4)		10.6 (9.1–12.2)	
Age				
22–34	4.9 (3.4–6.4)	0.20	6.0 (4.4–7.7)	< 0.001
35–44	5.0 (3.9–6.2)		8.2 (6.7–9.7)	
45–54	5.7 (3.9–7.5)		10.0 (7.6–12.3)	
55 or more	1.7 (0.0–3.7)		13.3 (8.4–18.7)	
Marital status				
Married or living together	4.2 (3.3–5.1)	0.08	7.2 (6.2–8.4)	< 0.001
Separated or divorced	6.8 (4.5–9.2)		10.8 (7.8–13.8)	
Widower	7.4 (1.7–13.1)		22.2 (13.1–31.3)	
Single	5.5 (3.7–7.3)		8.2 (6.0–10.5)	
Race/color				
Black	4.1 (2.3–5.9)	0.27	11.3 (8.4–14.2)	0.02
Brown	4.2 (2.9–5.4)		7.2 (5.5–8.8)	
White	5.9 (4.7–7.1)		8.0 (6.6–9.4)	
Asian	3.8 (0.0–8.9)		7.8 (0.4–15.3)	
Indigenous	3.1 (0.0–9.2)		18.7 (5.0–32.5)	
Education level				
Some elementary school	2.8 (0.6–5.1)	0.03	9.9 (5.8–14.0)	0.50
Elementary school	2.5 (0.5–4.6)		6.5 (3.3–9.7)	
Some high school	2.8 (0.9–4.7)		8.1 (4.8–11.4)	
High school	4.5 (2.9–6.0)		9.0 (6.9–11.2)	
Some college	6.7 (4.3–9.1)		7.4 (4.8–9.9)	
College	6.1 (4.5–7.8)		7.3 (5.5–9.1)	
Graduate	6.2 (3.5-8.8)		10.3 (7.0-13.7)	
Workplace				
UERJ Campus^1^	4.1 (3.1–5.1)	0.04	6.9 (5.6–8.2)	0.01
HUPE Hospital^2^	5.7 (4.6–6.9)		9.6 (8.1–11.1)	
Income equivalence (minimum wages)				
Up to 3	3.5 (1.3–5.8)	0.45	8.7 (5.1–12.3)	0.96
3 to 6	4.8 (3.3–6.3)		8.1 (6.2–10.1)	
>6	5.3 (4.3–6.3)		8.2 (7.0–9.5)	
Health insurance users				
Yes	5.6 (4.5–6.7)	0.10	8.7 (7.4–10.1)	0.32
No	4.2 (3.2–5.3)		7.7 (6.3–9.2)	
Alcohol consumption				
Yes	4.2 (3.2–5.2)	0.07	7.3 (6.0–8.6)	0.07
No	5.7 (4.4–6.9)		9.2 (7.6–10.8)	
Frequency of alcohol consumption (2 weeks)				
Every day	1.7 (0.0–5.0)	0.41	3.6 (0.0–8.6)	0.67
10 to 13 days	5.3 (0.0–11.3)		9.2 (1.4–17.0)	
6 to 9 days	7.2 (2.6–11.7)		8.1 (3.3–13.0)	
2 to 5 days	3.9 (2.6–5.2)		6.9 (5.2–8.7)	
1 single day	4.2 (2.5–6.0)		8.3 (5.9–10.7)	
Amount of alcohol consumed per day (2 weeks)				
1 standard unit	4.6 (2.7–6.5)	0.98	7.2 (4.8–9.5)	0.009
2 to 4 standard unit	4.1 (2.6–5.6)		5.8 (4.0–7.6)	
5 to 7 standard unit	4.1 (1.6–6.6)		13.0 (8.7–17.3)	
8 a 10 standard unit	3.8 (0.1–7.4)		7.9 (2.6–13.2)	
More than 10 standard unit	5.0 (0.7–9.4)		6.5 (1.4–11.6)	
Health condition				
Very good	3.6 (2.3–4.8)	< 0.001	5.1 (3.6–6.5)	< 0.001
Good	4.7 (3.7–5.7)		7.9 (6.6–9.2)	
Regular	8.0 (5.6–10.5)		14.2 (11.1–17.5)	
Bad	8.3 (0.0–17.5)		22.8 (8.7–37.0)	
Current health condition				
Better than 12 months ago	5.1 (3.3–6.9)	0.004	12.1 (9.4–14.9)	< 0.001
Same as 12 months ago	4.3 (3.5–5.2)		6.7 (5.6–7.8)	
Worse than 12 months ago	8.4 (5.6–11.2)		11.5 (8.1–14.9)	
GHQ-12 (Positive ≥ 3)				
Negative	3.7 (2.9–4.5)	< 0.001	6.3 (5.3–7.4)	< 0.001
Positive	8.0 (6.2–9.7)		12.7 (10.5–15.0)	
Difficulty falling asleep				
Always	10.6 (5.3–15.9)	< 0.001	21.7 (13.8–29.6)	< 0.001
Almost Always	4.6 (1.8–7.3)		21.8 (16.1–27.5)	
Sometimes	6.2 (4.4–8.0)		9.4 (7.2–11.2)	
Rarely	4.6 (3.0–6.2)		6.9 (5.0–8.9)	
Never	3.9 (2.8–5.0)		5.0 (3.8–6.2)	
Wake up during sleep cycle				
Always	11.5 (5.4–17.7)	< 0.001	19.5 (11.1–27.9)	< 0.001
Almost Always	3.4 (1.1–5.7)		19.2 (14.0–24.4)	
Sometimes	7.5 (5.5–9.5)		9.9 (7.6–12.3)	
Rarely	3.8 (2.4–5.3)		7.5 (5.5–9.5)	
Never	4.0 (2.9–5.0)		5.1 (3.5–6.4)	
Tiredness for no apparent reason				
Always	13.9 (8.0–19.7)	< 0.001	20.0 (12.8–27.2)	< 0.001
Almost Always	9.0 (5.8–12.2)		17.0 (12.7–21.3)	
Sometimes	6.2 (4.6–7.9)		9.8 (7.7–11.8)	
Rarely	2.8 (14.5–4.2)		4.8 (3.0–6.6)	
Never	3.0 (2.0–4.0)		5.2 (3.9–6.4)	

^1^ Universidade Estadual do Rio de Janeiro

^2^ Hospital University Pedro Ernesto

There was greater AD use among better educated individuals, whereas BDZ use was more uniform among the educational categories. The incidences of AD and BDZ use were 39% higher among individuals who worked at the university hospital. The incidence of AD and BDZ use was higher among those who reported having poor or regular health. Individuals who declared their health to be worse than it was 12 months before were shown to have higher incidence, although in BDZ cases this was also observed for those who declared their health to be better than before. The incidence of AD and BDZ use practically doubled among individuals with CMD. Most participants who started to use AD and BDZ after wave 1 reported sleep complaints and tiredness for no reason ( Table 3 ).

The incidence of AD use among BDZ users and non-users was investigated at the baseline and was three times higher among individuals who used BDZ (12.2%) compared with those who did not (4.6%).

### Multivariate models

#### Antidepressants

Women presented a cumulative incidence of AD use almost two and a half times higher than men in the studied period. Using the younger age group (22–34 years) as a reference, there was an increase of about 50% in the incidence in the 45–54 years age group, and a decrease by half in the group aged over 55, although the p *-* value reached a borderline level of significance (p = 0.06). The incidence of AD use for individuals with higher education, even if incomplete, was more than two times that for individuals without any higher education. Individuals suspected to have CMD (GHQ ≥ 3) had an AD use 34% higher than those with no disorder, but with a borderline p-value (0.08). The complaint of tiredness presented a dose-response relationship with the use of these psychotropic medicines ( Table 4 ).

**Table 4:** Poisson regression models for cumulative incidence of the antidepressants and benzodiazepines use. Pró-Saúde Study, Brazil.

Variable	Antidepressants		Benzodiazepines	
	
	RR (95%CI)	p-value	RR (95%CI)	p-value
Sex				
Men	1	< 0.001	1	0.001
Women	2.66 (1.75–4.04)		1.58 (1.3–2.1)	
Age				
22–34	1		NI	NI
35–44	1.15 (0.78–1.70)	0.47	NI	NI
45–54	1.54 (1.0–2.44)	0.062	NI	NI
55 or more	0.53 (0.16–1.77)	0.305	NI	NI
Marital status				
Married or living together	NI	NI	1	
Separated or divorced	NI	NI	1.20 (0.86–1.68)	0.28
Widower	NI	NI	2.15 (1.34–3.47)	0.002
Single	NI	NI	1.03 (0.82–1.55)	0.46
Race/Color				
Brown	NI	NI	1	
Black	NI	NI	1.47 (1.03–2.09)	0.03
White	NI	NI	1.26 (0.93–1.71)	0.14
Asian	NI	NI	1.25 (0.47–3.27)	0.65
Indigenous	NI	NI	3.15 (1.42–6.97)	0.005
Education level				
Some elementary school	1		NI	NI
Elementary school	0.99 (0.32–3.03)	0.98	NI	NI
Some high school	0.99 (0.34–2.84)	0.98	NI	NI
High school	1.7 (0.69–4.18)	0.25	NI	NI
Some college/ graduate	2.35 (0.98–5.62)	0.05	NI	NI
Health condition				
Very good	NI	NI	1	
Good	NI	NI	1.4 (0.98–2.00)	0.06
Regular/Bad	NI	NI	1.81 (1.2–2.72)	0.005
GHQ-12 (Positive ≥ 3)				
Negative	1	0.08	1	
Positive	1.34 (0.96–1.88)		1.34 (1.05–1.79)	0.03
Wake up during sleep cycle				
Never	NI	NI	1	
Rarely	NI	NI	1.23 (0.85–1.77)	0.27
Sometimes	NI	NI	1.45 (1.01–2.07)	0.04
Almost Always/Always	NI	NI	2.71 (1.86–3.94)	< 0.001
Tiredness for no apparent reason				
Never	1		NI	NI
Rarely	0.84 (0.47–1.50)	0.56	NI	NI
Sometimes	1.73 (1.11–2.70)	0.015	NI	NI
Almost Always	2.02 (1.20–3.42)	0.008	NI	NI
Always	3.2 (1.82–5.66)	< 0.001	NI	NI

NI – Not included in the final regression model since it presented a p-value > 0.10.

#### Benzodiazepines

Women presented a cumulative incidence 58% higher than men in the studied period. Individuals self-classified as brown, black, and indigenous individuals were shown to have higher risk of being a BDZ incident user. The incidence of these medicines among widowers was twice as high as that observed for married individuals. There was an increase in the incidence of BDZ use as the participants’ perception of their health status worsened. There was a 34% higher incidence of BDZ use among those with a GHQ-12 score compatible with CMD, as well as a direct dose-response pattern for the waking up during the sleep cycle variable ( Table 4 ).

## DISCUSSION

In the study population, an increase in both AD and BDZ use was observed between 1999 and 2012, although this increase has been proportionally higher for the first drug ( Figure 1 ). This pattern is consistent with what has been observed in other countries, having as probable causes the introduction of new AD pharmacological classes^1^ , the reduced side-effects of the most recent AD^1 , 2^ , and an expansion in the spectrum of AD indications^3 , 18^ .

This result partially confirms our initial hypothesis, as there was an increase in the use of AD, but not accompanied by a decrease in the BDZ use, but by more subtle increases in prevalence. That is, there was no substitution of BDZ by AD, but a possible association of both pharmacological classes to treat mental health disorders in some individuals^21^ .

Paulose-Ram et al.^22^ also reported an increase in the prevalence of AD (2.5%, 1988–1994 versus 8.1% 1999–2002) and BDZ use (3.5%, 1988–1994 versus 3.8%, 1999–2002) in the United States. However, a 1994 UK study found a reduction in the use of BDZ over time^23^ . This reduced use of BDZ was also reported by other authors^9^ .

A characteristic of the SSRI therapeutic approach is the recommendation of continuous and long-term treatment that may continue for up to 6 months or more after the symptoms disappear. It is possible the use of AD in our sample was continuous, while for BDZ it was more short-term and episodic (to deal with anxiety and insomnia episodes).

In this study, SSRI were the most used AD; the proportion of their users nearly doubled from 1999 to 2012, agreeing with many studies^14 , 24 , 25^ .

With the increase in SSRI use, there was a decrease in the use of other antidepressants, especially the TCA (mostly amitriptyline). The proportion of TCA users among AD users decreased by 75% between 1999 and 2012. This pattern agreed with other authors. In the US a reduction in the TCA use was observed, from 47% in 1987 to 2.1% by 2001^1^ . In Spain, in 2001–02, the frequency of SSRI use was 59.5%, and of TCA use was 25.5% among AD users^5^ . In the US, an increase in the prevalence of SSRI use was also observed; in 1999, the prevalence of SSRI use was 4.3%, increasing to 8.5% in 2012^25^ . In São Paulo, the prevalence of SSRI use was 2.17%, and of TCA was 1.26% in 2005–2007^11^ , while in Chile the prevalence of SSRI use was 2.0% and TCA use was 1.8% in 1996–98^4^ .

When examining the use of both psychotropic medicines, the incidence of BDZ use between 1999 and 2007 was twice as high as that for AD. In a longitudinal study conducted in France with less follow-up time than this study (1996–2001), it was found 2.3% of new AD users, 2.8% anxiolytics, and 2.3% hypnotics^26^ .

In this study, the first time an individual used AD or BDZ was not assessed, but the use that started (or restarted) during follow-up among those who did not use psychotropic medication at baseline was assessed. Since the assessment of incidence excluded AD and BDZ users at baseline, there may have been a higher chance of detecting the beginning or the return to BDZ use (medication that has a more cyclic or episodic use) than the beginning or return to AD use (a medication that tends to have a more prolonged and continuous use). This fact may have contributed to the higher increase in the found AD use compared with BDZ use in our follow-up.

Being a BDZ user at baseline increased the risk of the individual of starting AD use. The data are not surprising, given the evidence that primary care initiates the treatment of some mental disorders with BDZ before referring the individuals to specialists^3^ . The literature reports that the use of BDZ to treat depression is common and that this use may continue even after the introduction of AD^3^ . The use of AD was also found by Takeshima et al.^27^ to be a predictor not only for the initial use of BDZ but also as a predictor of prolonged used.

Cumulative incidence in the period from 1999 to 2006–07 was higher among women, both for AD and BDZ as others have observed^24 , 26^ . Anxiety disorders and depression have been consistently described as being more frequent among women^7 , 14^ , who also have a higher tendency to seek medical care^7^ ; besides, new indications for AD use encompasses ailments that are specifically or primarily suffered by women, such as premenstrual syndrome, premenstrual dysphoric disorder, eating disorders, fibromyalgia, and headaches^18 , 19 , 20 , 24^ .

There was an increase in the incidence of BDZ with age. However, in the AD case, the very oldest age group presented a significant reduction in incidence. This decrease in our sample may be due to a healthy worker effect^28^ ; the small size of this age group may have also affected the results.

Increased education level was associated with the incidence of AD use, but not with BDZ use. Some hypotheses may explain this result. Individuals with a greater education level may describe their condition more clearly, leading to a more adequate treatment. These individuals may seek help from a psychiatrist rather than a general practitioner^29^ ; furthermore, in general they have higher incomes, allowing them to afford the AD cost, usually higher than the BDZ cost.

The incidence of BDZ use was higher among individuals that classified their health status as poor, consistently with the fact that chronic health conditions, both physical and mental, increase the search for professional help and the prescription of psychotropic medicine^26 , 30^ . In the bivariate analysis, AD was also shown to be associated with the worst health perception. The fact that this association was not maintained in the multivariate analysis may be due to less statistical power in the analysis of these medicines, due to lower incidence when compared with BDZ. The incidence of BDZ use was also higher among people who reported having trouble falling and remaining asleep. This result is expected considering that BDZ is broadly prescribed worldwide for such conditions^27^ . However, complaints of tiredness in the baseline were associated with higher AD use, which can be explained because of this complaint association with depressive disorders.

This study reported the cumulative incidence of AD and BDZ use over a 13-year period, results that are unprecedented. Among the study limitations, the fact of not covering the medication use for the entire follow-up may have non-differentially misclassified some participants, therefore producing an underestimation of the incidence estimates. On the other hand, this restriction may have minimized the memory bias.

Contrary to our hypothesis and to the findings reported by other studies, this analysis did not corroborate the previous impression that the use of BDZ was being gradually replaced by the use of AD for many individuals. These results did not show an increase in the incidence of AD use followed by a decrease in the incidence of BDZ use. The reasons for such phenomenon are not clear. It is likely that AD is being added to the use of BDZ, and not just substituting the latter in the study population. It is imperative that AD and BDZ prescriptions are in agreement with the current treatment guidelines, especially considering the rational use of psychotropic medications.
